# Complete Genome Sequences of Four *Microbacterium* Strains Isolated from Metal- and Radionuclide-Rich Soils

**DOI:** 10.1128/MRA.00846-19

**Published:** 2019-10-17

**Authors:** Philippe Ortet, Nicolas Gallois, Justine Long, Séverine Zirah, Catherine Berthomieu, Jean Armengaud, Béatrice Alpha-Bazin, Mohamed Barakat, Virginie Chapon

**Affiliations:** a Aix-Marseille Université, CEA, CNRS, BIAM, Laboratoire des Interactions Protéine Métal, Saint-Paul-lez-Durance, France; b Aix-Marseille Université, CEA, CNRS, BIAM, Laboratoire d’Écologie Microbienne de la Rhizosphère et d’Environnements Extrêmes, Saint-Paul-lez-Durance, France; c Unité Molécules de Communication et Adaptation des Microorganismes (MCAM), Muséum National d’Histoire Naturelle, CNRS, Paris, France; d Laboratoire Innovations Technologiques pour la Détection et le Diagnostic (Li2D), Service de Pharmacologie et Immunoanalyse (SPI), CEA, INRA, Bagnols-sur-Cèze, France; University of Rochester School of Medicine and Dentistry

## Abstract

Here, we present the genome sequences of four *Microbacterium* strains, which were isolated at different locations in Europe from metal- or radionuclide-rich soils. High-quality complete genome sequences were obtained with PacBio and Illumina data sets with an original two-step procedure.

## ANNOUNCEMENT

The *Microbacterium* genus is composed of ubiquitous high-GC-content Gram-positive *Actinobacteria*. Despite its presence in many environments and its potential in bioremediation processes (1–7), the bacterial genus *Microbacterium* is underrepresented in the genome databases and is still poorly studied. At the time of writing, there were more than 100 *Microbacterium* species with a valid name (http://www.bacterio.net/microbacterium.html) and 323 genomes, including 28 complete genomes, available in the databases (https://www.ncbi.nlm.nih.gov/assembly/?term=microbacterium). Here, we report the complete genome sequences of four members of this genus, which were isolated from metal-rich or radionuclide-rich soil samples. The Microbacterium oleivorans strain A9 was isolated from radionuclide-contaminated soil in Chernobyl (8) and exhibits a high uranium tolerance (9). Its draft genome sequence was previously published (10). The strains ViU2A and ViU22^T^ were isolated from natural uranium-rich soil samples collected in France (the soil composition is described in reference 11; the strain ViU22^T^ is the type strain of the species Microbacterium lemovicicum [12]). The strain HG3 was cultured from metal-rich black sand from Iceland and has been established as mercury tolerant (13).

Bacteria were cultured in LB at 30°C until late exponential growth phase. High-quality genomic DNA was extracted from cells using the DNeasy blood and tissue kit (Qiagen) following the manufacturer’s instructions for Gram-positive bacteria. DNA integrity was checked using agarose gel electrophoresis, and DNA purity and concentration were measured using a NanoDrop spectrophotometer (Thermo Fisher Scientific). Whole-genome shotgun sequencing was carried out with PacBio long-read technology and the Illumina short-read technology. Library preparation and single-end sequencing with 100-base read lengths and a HiSeq 2000 instrument (Illumina) were performed by GenoScreen (Lille, France). Library preparation and long-read sequencing were performed by Eurofins Genomics Europe Shared Services GmbH (formerly GATC Biotech AG, Germany). Library preparation incorporated adaptor sequences compatible with PacBio RS II sequencing technology (single-molecule real-time [SMRT] sequencing) using proprietary methods of Eurofins Genomics Europe Shared Services GmbH. Sequencing was carried out on a PacBio RS II system with SMRT technology. For both sequencing methods, the total number of reads is indicated in Table 1.

**TABLE 1:** Summary of complete genome sequences

Characteristic	Data for strain:			
	A9	Hg3	ViU2a	ViU22
GenBank accession no.	CP031421	CP031422	CP031338	CP031423
Raw data accession no. (Illumina)	SRR8416123	SRR8416223	SRR8416225	SRR8417352
No. of reads (Illumina)	18,500,154	19,895,949	15,648,367	13,317,740
Raw data accession no. (PacBio)	SRR8416122	SRR8416222	SRR8416224	SRR8417351
No. of reads (PacBio)	144,950	154,761	172,715	164,482
Mean read length (bp) (PacBio)	8,474	8,380	7,755	8,270
Genome length (Mb)	2.99	3.9	3.8	3.6
G+C content (%)	69	68.2	68.3	70.8
No. of protein-coding genes	2,880	3,764	3,708	3,297
No. of tRNA genes	50	51	54	52
No. of rRNA genes	6	6	6	6
No. of TCSs	62	70	75	55
No. of TFs	156	274	280	191

In the first step, genome *de novo* assembly was performed on the PacBio reads using Canu software version 1.0 with default parameters (14). Trimming and circularization in a single genome assembly were done using this tool. Then, to improve the quality of the genome sequence, a correction was made using the Illumina reads with the MIRA assembler version 4.0.2 (15) on the complete genome sequence (assembled using Canu and oriented on the origin of replication [*oriC*] by an in-house pipeline) as reference. We used the default configuration for MIRA except for the option job, which was set to “genome,” “mapping,” and “accurate,” and for the option parameters, which were set to SOLEXA_SETTINGS -CL:pec=yes COMMON_SETTINGS -NW:cac=no -SK:mmhr=1.

The accession numbers, assembly metrics, and genome characteristics for each genome are listed in Table 1. All chromosomal sequences were circularized and oriented with the predicted *oriC* region as the beginning of the sequences. Taxonomic assignment at the species level was provided by the NCBI using their quality control test for bacterial genomes. This test uses average nucleotide identity (ANI), which compares the submitted genome sequence against the genomes of the type strains and proxytype strains that are already in GenBank, as described in reference 16. The chromosomal sequences were annotated with the NCBI Prokaryotic Genome Annotation Pipeline version 1.2 with default parameters (17). Two-component system proteins (TCSs) and transcription factor proteins (TFs) were identified using the P2RP Web server version 2.7 with default parameters (18).

### Data availability.

The raw data and whole-genome sequences of the four *Microbacterium* strains have been deposited in the GenBank database under the accession numbers listed in Table 1.
